# Circulating endothelial progenitor cells and large artery structure and function in young subjects with uncomplicated Type 1 Diabetes

**DOI:** 10.1186/1475-2840-10-88

**Published:** 2011-10-08

**Authors:** Carlo Palombo, Michaela Kozakova, Carmela Morizzo, Laura Gnesi, Maria Chiara Barsotti, Paolo Spontoni, Francesco Massart, Paolo Salvi, Alberto Balbarini, Giuseppe Saggese, Rossella Di Stefano, Giovanni Federico

**Affiliations:** 1 Department of Surgery, University of Pisa, via Paradisa 2, Pisa, 56 124, Italy; 2 Cardiac, Thoracic and Vascular Department, University of Pisa, via Paradisa 2, Pisa, 56124, Italy; 3 Department of Reproductive Medicine and Child Development, Division of Pediatrics, University of Pisa, via Roma 67, Pisa, 56126, Italy; 4 Istituto Auxologico IRCCS, S. Luca Hospital, piazalle Brescia 20, Milano, 20149, Italy

**Keywords:** Type 1 diabetes, Endothelial progenitor cells, Endothelium-dependent vasodilation, Radiofrequency based ultrasound, Carotid intima-media thickness, Carotid stiffness, Aortic stiffness, Arterial wave reflection, Advanced glycation end-products, Adiponectin

## Abstract

**Background:**

Carotid intima-media thickness (IMT), indices of large artery stiffness and measures of endothelium function may be used as markers of early atherosclerosis in type 1 diabetes mellitus (T1DM). The aim of the present study was to compare the indices of large artery structure and function as well as endothelial function and regenerating capacity between adolescents with T1DM and healthy control of similar age. In addition, the associations of different vascular measures with endothelial progenitor cells (EPCs), glyco-metabolic control and serum levels of advanced glycation endproducts (AGEs), soluble receptors for AGEs (sRAGE) and adiponectin were evaluated.

**Methods:**

Sixteen uncomplicated young T1DM patients (mean age 18 ± 2 years, history of disease 11 ± 5 years, HbA1c 7.7 ± 1.1%) and 26 controls (mean age 19 ± 2 years) were studied. A radiofrequency-based ultrasound system (Esaote MyLab 70) was used to measure carotid IMT and wave speed (WS, index of local stiffness), applanation tonometry (PulsePen) was applied to obtain central pulse pressure (PP) and augmentation index (AIx), and carotid-femoral pulse wave velocity (PWV, Complior) was used as index of aortic stiffness. Peripheral endothelium-dependent vasodilation was determined as reactive hyperemia index (RHI, EndoPAT). Circulating EPCs, glycometabolic profile, AGEs (autofluorescence method), sRAGE and adiponectin were also measured.

**Results:**

After adjusting for age, sex and blood pressure, T1DM adolescents had significantly higher carotid IMT (456 ± 7 vs. 395 ± 63 μm, p < 0.005), carotid WS (p < 0.005), PWV (p = 0.01), AIx (p < 0.0001) and central PP (p < 0.01) and lower EPCs (p = 0.02) as compared to controls. RHI was reduced only in diabetic patients with HbA1c ≥7.5% (p < 0.05). In the overall population, EPCs were an independent determinant of carotid IMT (together with adiponectin), while fasting plasma glucose was an independent determinant of carotid WS, AIx and central PP.

**Conclusions:**

Our findings suggest that young subjects with relatively long-lasting T1DM have a generalized preclinical involvement of large artery structure and function, as well as a blunted endothelium regenerating capacity. Hyperglycemia and suboptimal chronic glycemic control seem to deteriorate the functional arterial characteristics, such as large arteries stiffness, wave reflection and peripheral endothelium-dependent vasodilation, whereas an impaired endothelium regenerating capacity and adiponectin levels seem to influence arterial structure.

## Background

Type 1 diabetes mellitus (T1DM) is a major risk factor for cardiovascular disease as the incidence of cardiovascular complications in T1DM patients is reported to be 2- to 10-fold higher than in normal population [1]. Clinically overt diabetes-related vascular complications are rare in childhood and adolescence, and an optimal glycemic control at earlier age and stage of the disease may attenuate the development and progression of functional and structural alterations in the arterial tree [2,3]. It is therefore important to identify preclinical vascular changes at a very early stage in order to improve glycemic control and reduce the risk of later cardiovascular complications. A number of noninvasive measurements, such as endothelium-dependent flow-mediated vasodilation (FMD), common carotid artery intima-media thickness (C-IMT), carotid-femoral pulse velocity (PWV, an index of aortic stiffness) and carotid augmentation index (AIx, an index of arterial pressure wave reflection) have been proposed as "tissue biomarkers" capable to improve risk stratification and tracking early atherosclerotic disease beyond the simple determination of established risk factors [4].

Endothelial dysfunction has been suggested to anticipate structural changes in large artery wall in young T1DM patients [5], in keeping with the hypothesis that functional abnormalities of the endothelium represent not only an early marker of atherosclerosis but above all a pathophysiologic mechanism promoting the development of arterial wall thickening and stiffening [6]. This hypothesis was further confirmed in a recent study on young T1DM patients [7] that demonstrated a severe impairment of FMD, important reduction in bone marrow derived circulating endothelial progenitor cells (EPCs), moderate increase in C-IMT and an inverse relationship between FMD and C-IMT. C-IMT has been extensively evaluated in children and adolescents with T1DM, and an increased C-IMT has been reported not only in subjects with suboptimal metabolic control [8] but also in those with satisfactory insulin treatment and with a relatively short diabetes duration (5.5 years) [9-11]. On the other hand, reports on large artery stiffness in young diabetic patients are still limited and indirect, based mainly on applanation tonometry [12,13].

Various metabolic abnormalities with atherogenic potential, like fasting and post-prandial high plasma glucose levels, increased advanced glycated endproducts (AGEs) and post-secretory modified LDL particles, may be accountable for accelerated development and progression of vascular organ damage in T1DM [14-17]. The possible role of adiponectin in T1DM-related vascular changes [18] requires further elucidation, as plasma levels of this atheroprotective adipokine have been reported increased in young peoples with T1DM [19,20].

To identify a sensitive and early marker of vascular organ damage in T1DM and to provide some insight on mechanisms underlying these early vascular changes, the present study compared several different indices of vascular structure and function between T1DM adolescents and healthy controls of similar age, and it also tested the relationships between the vascular measures and circulating EPCs, glyco-metabolic control, serum levels of AGEs, soluble receptors for AGEs (sRAGE) and adiponectin.

## Methods

### Study population

We studied 16 consecutive subjects with T1DM and 26 healthy controls. The diabetic population consisted of outpatients with a relatively long history of disease (11 ± 5 years) undergoing periodic follow-up examinations at the Pediatric Endocrinology and Diabetes Unit, Pediatrics Department, University of Pisa. At the time of the study, they were all taking a combination of rapid-acting, basal insulin analogs (basal-bolus regimen, optimized according to clinical evaluation and HbA1c). In all patients, the presence of concomitant disease (other than diabetes mellitus), pharmacological treatment (other than insulin), or cigarette smoking, alcohol, or food abuse was excluded by medical history, standard medical examinations and clinical tests. All subjects were free from overt macrovascular complications, microalbuminuria, retinopathy and neuropathy. The control group of 26 healthy subjects consisted of friends of the diabetic patients studied and children and adolescents of Pisa University Hospital staff members. All females, when fertile, were investigated during the follicular phase of menstrual cycle.

### Study protocol

The protocol of the study followed the principles of the Declaration of Helsinki and was approved by the institutional ethics committee. All patients (or a parent if minor) gave their informed consent to participate. Each study participant underwent an integrated vascular examination, two hours after a light breakfast free of caffeine containing beverages. The same day, venous blood samples were taken after an overnight fast, between 07.30 and 08.30 am, in order to measure blood glucose, HbA1c, serum lipids, circulating EPCs, sRAGE and total circulating adiponectin.

### Analytical procedures

Blood glucose, total cholesterol and its fractions, and triglycerides were assayed with standard laboratory methods. HbA1c was assessed with HPLC (G7 HPLC Analyzer, Tosoh Bioscience Inc, South San Francisco, USA; reference range 4.3-6.1%). Circulating total adiponectin was measured with a specific radioimmunoassay (DRG Diagnostics International Inc, Mountainside, USA; sensitivity of 1 ng/ml, an intra- and inter-assay coefficients of variation of 3.6% and 6.9%, respectively). Circulating soluble receptors of AGEs (sRAGE) levels were assayed using a commercial ELISA kit (DuoSet ELISA development kit; R&D Systems, Minneapolis, U.S.A.; sensitivity of 21.5 pg/ml, an intra- and interassay coefficients of variation < 6.0% and < 8.5%, respectively).

### AGE-dependent skin autofluorescence

AGE-dependent skin autofluorescence (AF) was measured by the AGE Reader apparatus (DiagnOptics, Groningen, the Netherlands). In accordance with the manufacturer's instructions, measurements were taken at three different sites on the inner side of the arm, with the subject in a seated position. AF was expressed in arbitrary units [21].

### Quantification of peripheral blood endothelial progenitor cells (EPCs)

EPCs were analyzed for the expression of surface antigens with direct two-color flow cytometry (FACS Calibur; BD Biosciences, Franklin Lakes, NJ, USA). A 100 μL sample of peripheral blood was stained with 10 μL of PERcP-conjugated human anti-CD34 monoclonal antibody (mAb) (BD Biosciences) and 10 μL fluorescein-conjugated anti-human kinase insert domain receptor (KDR) mAb (R&D Systems, Minneapolis, MN, USA) or isotype control for 30 minutes in the dark at 4°C. Red cells were lysed with 1x BD FACS Lysis solution (BD Pharmigen, UK) and incubated for 5 minutes at room temperature. Samples were analyzed on a BD FACS Calibur flow cytometer with CellSystems^® ^software (Becton Dickinson, UK). The frequency of peripheral blood cells positive for the above reagents was assessed with a two-dimensional side scatter fluorescence dot plot analysis, after appropriate gating and staining with the different reagents: we initially gated CD34^+ ^peripheral blood cells in the mononuclear cell fraction and then examined the resulting population for dual expression of KDR. Validation of the assay was carried out by the ISHAGE method. For FACS analysis, 5 × 10^5 ^cells were acquired. CD34+ cell counts were expressed as a percentage of the total gated leukocytes population; CD34+KDR+ cells (EPCs) were expressed as percentage of total CD34+ cells. The operator who carried out all the tests (MC.B.) was trained in flow cytometry, experienced in rare event analysis and unaware of the participants' clinical status.

### Integrated vascular investigation

The integrated vascular investigation was performed in a quiet room, with a stable temperature of 22°, starting with carotid scanning, after resting comfortably for at least 15 minutes in supine position. A single operator (C.M.) unaware of the clinical status of the subject under investigation performed all vascular recordings and readings.

#### Carotid Ultrasound

Carotid ultrasound was performed on the right carotid artery using an ultrasound scanner equipped with a linear 10 MHz probe (MyLab 70, Esaote, Genova, Italy) and implemented with a previously validated radiofrequency-based tracking of arterial wall that allows a real-time determination of common carotid far-wall thickness (QIMT^®^) and distension (QAS^®^) with high spatial and temporal resolution [22]. Far-wall IMT (C-IMT) was automatically measured, and distension curves were acquired within a carotid segment ~ 1 cm before the flow divider, where the operator placed the region of interest. From the distension curves, maximum and minimum carotid diameters were obtained and indices of vascular stiffness were calculated after calibration for blood pressure (BP). In this study, we used a "local" carotid wave speed (WS), calculated from beta-stiffness index (β) and local pulse pressure (PP) according to the following equations:

1) β stiffness index = ln (systolic BP/diastolic BP)/ΔD/D, where ΔD is systo-diastolic distension and D is the end-diastolic diameter [23], and:

2) WS (m/s) = PP/2* β, where PP is local carotid pulse pressure.

BP was measured at the left brachial artery (Omron, Kyoto, Japan) during the acquisition of the distension curves, and brachial PP was rescaled to obtain local carotid PP from a simultaneously recorded carotid arterial waveform by applanation tonometry (see below) [24]. All measures were averaged over 6 consecutive cardiac beats and the values used for statistical analysis represent a mean of three acquisitions. In our laboratory intra-individual variability of QIMT and QAS measurements are 3.5 ± 1.9 and 5.8 ± 3.4%, respectively.

#### Carotid Artery Applanation Tonometry

Carotid applanation tonometry was performed on the left CCA with a validated system (PulsePen, Diatecne, Milan) [25] simultaneously to the ultrasound investigation, in order to obtain local PP and AIx, an index of arterial wave reflection from the periphery [26]. For statistical analysis, values of AIx averaged through 5 consecutive heart beats were used. In our laboratory intra-individual variability of PP and AIx measurements are 4.2 ± 1.7 and 4.9 ± 3.1%, respectively.

#### Carotid-Femoral Pulse Wave Velocity (PWV)

Carotid-femoral PWV was measured according to current guidelines [27]. Briefly, arterial waveforms were obtained over the right CCA and femoral artery, and the time delay (t) was measured between the feet of the two waveforms. The distance (D) covered by the waves was established as the distance between the two recording sites. PWV was then calculated as D (meters)/t (seconds). The measurement was performed three times, using the Complior device (Alam Medical, Vincennes, France) and the mean value was used for statistical analysis. Simultaneous BP measurement was performed at the left brachial artery (Omron, Kyoto, Japan). In our laboratory, intra-individual variability of PWV measurement is 4.5 ± 2.8%.

#### Endothelium-Dependent Vasodilation

Peripheral endothelium-dependent vasodilator capacity was estimated assessing a reactive hyperemia index (RHI) by means of an EndoPAT system (Itamar Medical Ltd, Cesarea, Israel) [28]. Briefly, the subject sat in a reclining chair with the hands at heart level and was propped in a comfortable position so that the fingers were hanging freely. Fingertip probes were placed on both index fingers and pulse wave amplitudes were recorded for the duration of the entire study that consists of: 5-minute baseline recording; 5-minute occlusion of non-dominant arm using a BP cuff inflated to 40 mmHg above systolic pressure; rapid deflation of BP cuff (followed by reactive, flow-mediated hyperemia) and pulse wave amplitudes recording for at least further 5 minutes. An integrated software program compares the ratio of arterial pressure waves in the two fingers before the occlusion and after the deflation to calculate the reactive RHI score as the ratio of the average pulse wave amplitude, measured over 60 seconds starting one min after cuff deflation, to the average pulse wave amplitude measured at baseline. This ratio is normalized to the concurrent signal from the contralateral finger to correct for changes in systemic vascular tone.

### Statistical analysis

Data are expressed as mean ± SD. Variables with a skewed distribution are given as median [interquartile range] and were log transformed for use in statistical analyses. ANOVA was used to compare continuous variables between T1DM patients and controls. Relationships between vascular measurements and metabolic parameters were evaluated by univariate Pearson's correlation coefficients. Multiple regression analysis was then used to test the independence of these associations. Statistical tests were two-sided and significance was set at p < 0.05. Statistical analysis was performed by JMP software, version 9.01 (SAS Institute Inc, Cary, NC, USA).

## Results

The characteristics of study population are described in Table 1. After adjustment for sex and age, T1DM and controls differed only in plasma fasting glucose and not in plasma lipids, hemodynamic parameters or plasma adiponectin level. EPCs were significantly decreased in T1DM, but circulating sRAGEs as well as skin autofluorescence values were similar in T1DM and controls. Mean HbA1c value in our T1DM patients was 7.7 ± 1.1% (range from 5.8 to 10.3%).

**Table 1:** Clinical and Metabolic Characteristics of Study Population

	Controls	T1DM	p
N	26	16	
Sex (males, %)	58	68	= 0.47
Age (years)	19 ± 2	18 ± 2	= 0.12
Body mass index (kg/m^2^)	21.6 ± 4.2	21.7 ± 6.2	= 0.82
Systolic BP (mmHg)	112 ± 10	118 ± 16	= 0.13
Diastolic BP (mmHg)	67 ± 5	66 ± 7	= 0.94
Mean BP (mmHg)	82 ± 5	83 ± 8	= 0.47
Heart rate (bpm)	70 ± 12	69 ± 11	= 0.88
Total cholesterol (mg/dL)	165 ± 22	170 ± 33	= 0.37
HDL-cholesterol (mg/dL)	58 ± 13	54 ± 8	= 0.36
LDL-cholesterol (mg/dL)	87 ± 19	100 ± 21	= 0.08
**^∫^**Triglycerides (mg/dL)	71 [31]	75 [25]	= 0.94
**^∫^**Adiponectin (mg/L)	18.5 [5.5]	19.0 [4.5]	= 0.09
***Fasting plasma glucose (mg/dL)***	***87 ± 7***	***178 ± 45***	***< 0.0001***
sRAGEs (pg/ml)	383 ± 152	464 ± 187	= 0.76
AGE-dependent AF (arbitrary units)	1.6 ± 0.3	1.7 ± 0.3	= 0.52
^∫^EPCs (CD34+/KDR+/10^6 ^events*)	***74 [150]***	***39 [63]***	***= 0.02***

**^∫^**: skewed variables expressed as median [interquartile] range; * EPCs: log-transformed endothelial progenitor cells expressed as CD34+/KDR+ cells/10^6 ^cytometric events; p values after adjustment for sex and age. Statistically significant differences are highlighted in bold italic.

**Figure 1:**
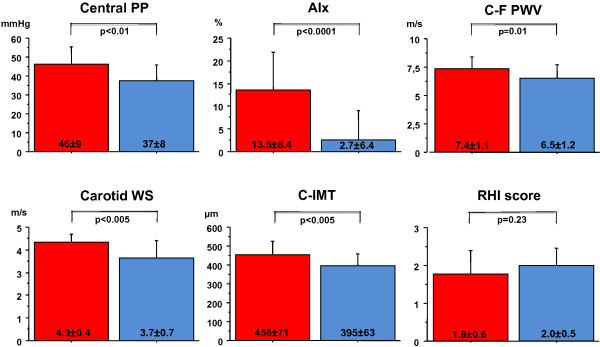
**Indices of vascular structure and function in young T1DM patients and healthy controls**. Central pulse pressure (PP), augmentation index (AIx), carotid-femoral pulse wave velocity (C-F PWV), local carotid wave speed (Carotid WS), common carotid artery intima-media thickness (C-IMT), and reactive hyperemia index (RHI score) in subjects with type 1 diabetes (red) and controls (light blue). Means ± sd are presented. P values are reported after adjustment for sex, age and mean BP.

Vascular parameters in T1DM and controls are reported in Figure 1. After adjustment for sex, age and mean BP, T1DM adolescents had higher central PP, AIx, carotid-femoral PWV as well as local carotid WS and C-IMT. Peripheral endothelium-dependent vasodilator capacity (RHI score) did not differ significantly between the 2 groups, although it was lower in T1DM patients with HbA1c ≥7.5% as compared to those with HbA1c < 7.5% (1.5 ± 0.4 vs. 2.2 ± 0.8, p < 0.05) and decreased with diabetes duration (r = -0.54, p < 0.05).

To identify the metabolic variables accounting for significant differences in vascular parameters between healthy and T1DM young subjects (Figure 1) we tested, in the entire study population, the univariate associations of vascular measures with plasma glucose, lipids, adiponectin, AGEs, sRAGEs and EPCs (Table 2). Subsequently, multiple regression analyses were performed, entering standardized vascular measures as dependent variables, and sex, age, mean BP and all metabolic correlates (in univariate analyses) as independent variables (Table 3).

**Table 2:** Univariate Pearson Correlation Coefficients Between Vascular Measures and Metabolic Parameters

	Central PP (mmHg)	AIx (%)	C-F PWV (m/s)	Carotid WS (m/s)	C-IMT (μm)	RHI score
**Fasting plasma glucose**	***r = 0.36***	***r = 0.47***	***r = 0.33***	***r = 0.42***	r = 0.29	r = -0.26
**(mg/dL)**	***(p < 0.05)***	***(p < 0.005)***	***(p < 0.05)***	***(p < 0.01)***	(p = 0.06)	(p = 0.10)

**HDL-cholesterol**	***r = -0.40***	r = -0.17	r = -0.16	r = -0.09	***r = -0.30***	r = 0.24
**(mg/dL)**	***(p = 0.01)***	(p = 0.31)	(p = 0.33)	(p = 0.59)	***(p = 0.05)***	(p = 0.14)

**LDL-cholesterol**	r = 0.06	r = 0.06	***r = 0.35***	r = 0.26	r = 0.04	r = -0.05
**(mg/dL)**	(p = 0.71)	(p = 0.72)	***(p < 0.05)***	(p = 0.11)	(p = 0.80)	(p = 0.77)

***^∫^*Adiponectin**	r = -0.13	***r = -0.44***	r = -0.01	r = -0.08	***r = -0.36***	r = 0.24
**(mg/L)**	(p = 0.47)	***(p < 0.01)***	(p = 0.96)	(p = 0.65)	***(p < 0.05)***	(p = 0.16)

***^∫^*EPCs**	r = 0.08	r = -0.27	r = -0.12	r = -0.12	***r = -0.31***	r = 0.20
(CD34+/KDR+/10^6 ^events*)	(p = 0.63)	(p = 0.10)	(p = 0.45)	(p = 0.46)	***(p < 0.05)***	(p = 0.21)

**^∫^**: skewed variables log transformed; significant correlations are highlighted in bold italic

**Table 3:** Independent Correlates of Vascular Parameters: Multiple Regression Model *(β = standardized regression coefficient).

		*β ± SE	p
**Central PP (mmHg)**			
	***Sex (male)***	***0.57 ± 0.15***	***p < 0.0005***
	Age (years)	-0.07 ± 0.13	p = 0.60
	Mean BP (mmHg)	0.05 ± 0.13	p = 0.69
	HDL-cholesterol (mg/dL)	-0.01 ± 0.13	p = 0.96
	***Fasting glucose (mg/dL)***	***0.29 ± 0.12***	***p = 0.01***
*Cumulative R^2 ^*		*0.54*	*p < 0.0001*

**AIx (%)**			
	***Sex (male)***	***0.34 ± 0.16***	***p < 0.05***
	Age (years)	0.01 ± 0.15	p = 0.98
	Mean BP (mmHg)	-0.11 ± 0.16	p = 0.53
	***Fasting glucose (mg/dL)***	***0.44 ± 0.14***	***p < 0.005***
	***^∫^Adiponectin (mg/L)***	***-0.49 ± 0.14***	***p < 0.005***
*Cumulative R^2^*		*0.51*	*p = 0.0001*

**C-F PWV (m/s)**			
	***Sex (male)***	***0.39 ± 0.16***	***p < 0.05***
	Age (years)	0.30 ± 0.16	p = 0.06
	Mean BP (mmHg)	0.07 ± 0.15	p = 0.66
	***LDL-cholesterol (mg/dL)***	***0.36 ± 0.16***	***p < 0.05***
	Fasting glucose (mg/dL)	0.24 ± 0.19	p = 0.13
*Cumulative R^2^*		*0.35*	*p < 0.01*

**Carotid WS (m/s)**			
	Sex (male)	0.24 ± 0.16	p = 0.15
	Age (years)	0.26 ± 0.16	p = 0.13
	Mean BP (mmHg)	0.10 ± 0.16	p = 0.55
	***Fasting glucose (mg/dL)***	***0.46 ± 0.16***	***p < 0.01***
*Cumulative R^2^*		*0.29*	*p < 0.05*

**C-IMT (μm)**			
	***Sex (male)***	***0.49 ± 0.17***	***p < 0.01***
	***Age (years)***	***0.39 ± 0.14***	***p < 0.01***
	Mean BP (mmHg)	0.14 ± 0.14	p = 0.30
	HDL-cholesterol (mg/dL)	-0.20 ± 0.15	p = 0.19
	**^∫^*Adiponectin (mg/L)***	***-0.35 ± 0.13***	***p = 0.01***
	***^∫^EPCs***	***-0.48 ± 0.13***	***p = 0.001***
*Cumulative R^2 ^*		*0.57*	*p < 0.0005*

**^∫^**: skewed variables log transformed; independent correlates are highlighted in bold italic.

In univariate analysis, with increasing fasting plasma glucose increased central PP, AIx, C-F PWV and carotid WS. With increasing HDL-cholesterol decreased central PP and C-IMT, whereas with increasing LDL-cholesterol increased C-F PWV. With increasing plasma adiponectin decreased AIx and C-IMT, which was also inversely related to EPCs (Table 2). Triglycerides, AGEs and sRAGEs did not correlate with any parameter of vascular structure or function. In successive multivariate models, fasting plasma glucose resulted as an independent determinant of central PP, AIx and carotid WS. LDL-cholesterol was an independent determinant of C-F PWV. Adiponectin was an inverse and independent correlate of AIx and C-IMT, and EPCs were inversely and independently correlated with C-IMT (Table 3). When diagnosis of T1DM was included into the multiple regression model of central PP, AIx and carotid WS, it replaced fasting plasma glucose as an independent determinant of these vascular measures. In regression model of C-IMT, T1DM diagnosis replaced EPCs and adiponectin.

We also evaluated the possible inter-relationships between structural and functional vascular measures. In the entire study population, C-IMT was directly related to AIx, C-F PWV and carotid WS (r = 0.51, 0.30 and 0.31, respectively, p at least < 0.05), and inversely to RHI score (r = -0.34, p < 0.05)

## Discussion

In recent years, particular attention has been paid to the preclinical alterations in the vascular system in diabetic children and adolescents in order to prevent development of overt atherosclerosis and clinical events in the adulthood. The high risk of atherosclerosis in diabetes is mainly attributed to endothelial dysfunction that results both from endothelial cell damage and impaired endothelial repair [29]. Bone-marrow derived circulating EPCs are believed to contribute to endothelial repair, to vascular homeostasis and to compensatory angiogenesis [30], and their decrease in diabetes was shown to closely correlate with the severity of carotid atherosclerosis and progression of diabetic complications [31,32]. Endothelial dysfunction is considered not only the first marker of vascular damage but also a pathophysiologic mechanism promoting further structural changes within the arterial wall [6].

The results of our study confirm and extend this hypothesis. Our young T1DM patients free of overt clinical complications had, as compared to controls of similar age, a significantly reduced number of specific CD34^+^KDR^+ ^EPCs, and, in entire study population, EPCs count was inversely and independently related to C-IMT. These findings are in agreement with previous data in adults [7,33-35], as well as with assumptions that the abnormalities of endothelial function precede the development of clinically evident arterial damage and that an impaired regenerative capacity of the endothelium yields an increased risk of developing early atherosclerotic changes [35]. Together with a low EPCs count, a low plasma adiponectin was another independent determinant of increased C-IMT in our young population. An inhibitory effect of adiponectin against excessive intimal growth was previously reported in experimental carotid injury [36], and a large cross-sectional study in healthy subjects has proposed a low adiponectin as a risk factor for early atherosclerosis [37]. Although our data confirmed a protective role of adiponectin against early carotid atherosclerosis, they did not confirm the increase in plasma adiponectin levels in T1DM patient that has been described by other authors [19,20]. This discrepancy can be explained by a better glycemic control of our patients, since Barnes el al. [20] have demonstrated, in a large population of 440 young T1DM subjects, a positive correlation between adiponectin and HbA1c. Mean HbA1c of our patients (7.7%) was lower that that of young T1DM subjects (9.8%), in which an increase in plasma adiponectin levels has been demonstrated [19].

Chronic glycemic control might also affect endothelial function. Although RHI score, an index of peripheral endothelium-dependent vasodilator capacity, was not decreased in our T1DM patients as compared to controls, it was significantly lower in patients with HbA1c ≥7.5% than in those with HbA1c < 7.5%. Furthermore, the glycemic control of T1DM children and adolescents in studies that described an impairment of brachial artery flow-mediated dilation and microvascular flow-mediated vasodilatation [7,38,39] was worse than in our group (HbA1c 8.9, 8.5 and 9.4 vs. 7.7%).

Data on large artery stiffness in children and adolescents with T1DM are more sporadic and report above all an increase in AIx [12,13]. AIx is an integrated measure of stiffness and reflection from the periphery, and thus it does not represent a proper estimate of large artery function [26]. It has been suggested that an augmented pressure wave reflection in diabetes is a consequence of combined changes of both small resistance arteries and large arteries [40]. Increased radial artery AIx has been demonstrated in T1DM children as young as 10 years of age [12], and increased carotid artery AIx has been observed in children and adolescents with relatively short duration of diabetes (5.4 ± 3.4 years) and poor glycemic control (HbA1c 9.8%) [41]. Recent data from the SEARCH of Diabetes in Youth Study have confirmed an increased AIx in T1DM children together with a decrease of brachial artery distensibility [42]. In our study, young T1DM patients showed increased local carotid stiffness, aortic stiffness and wave reflection, which together resulted in a significantly higher central pulse pressure despite similar blood pressure values at brachial artery level. In addition, in the entire study population, local carotid stiffness, AIx and pulse pressure were independently associated with fasting plasma glucose, a finding that confirms data regarding the effect of acute hyperglycemia on arterial stiffness of T1DM patients [43] and emphasizes the importance of a strict glycemic control. Interestingly, carotid-femoral PWV was not independently related to plasma glucose but to LDL-cholesterol. The different impact of metabolic factors on aortic and carotid stiffness implies that the two vascular districts may be not interchangeable in predicting cardiometabolic risk [44].

It must be also taken in consideration that increased local carotid thickness, stiffness and wave reflection may ultimately results in a positive feedback loop, promoting further progression of cardiovascular disease. The altered local mechanical properties of the carotid artery could play a role in a baroreflex gain reduction that may increase the susceptibility to develop hypertension [45], and an increased central pulse pressure may represent a mechanism accelerating intima-media thickening over the time [46].

### Study limitations

The present study has some limitations. A relatively small number of subjects included in the two groups does not exclude that the study could have been underpowered for some of the subgroup analyses, and it also did not allow performing the regression analyses between vascular indices and metabolic variables separately for T1DM patients and controls. We did not determine AGEs directly on plasma assays, but used a surrogate noninvasive estimate based on skin autofluorescence.

## Conclusions

The results of this study suggest that young subjects with relatively long-lasting T1DM and free of overt clinical complications, have a significantly lower count of circulating EPCs and a generalized preclinical involvement of large artery structure and function as compared to healthy controls. An increase in C-IMT is independently related to decrease in circulating EPCs and to lower plasma adiponectin, whereas impairment of peripheral flow-mediated dilation seems to be associated with worse chronic glycemic control. Local carotid stiffness and pressure wave reflection are mainly determined by fasting plasma glucose, while aortic stiffness is depending on plasma LDL-cholesterol. From a clinical point of view, our findings highlight the need of a more strict control of glycemic levels, considering also the fact that hyperglycemia represents a primary contributor to the reduced number and impaired function of EPCs in diabetes [18,30]. Although our T1DM young patients had a better glycemic control than that found in the adolescent cohort of the Diabetes Control and Complications Trial (a mean HbA1c of 8.1%,) [3], various patients showed an average HbA1c still above the recently indicated target of HbA1c below 7.5% [47]. Finally the radiofrequency-based tracking of arterial wall, which allows an accurate real-time determination of the basic blood vessel wall properties, may facilitate and anticipate the detection of early vascular organ damage in young T1DM subjects [48].

## List of abbreviations

AF: skin autofluorescence; AGEs: advanced glycation endproducts; Aix: carotid augmentation index; BP: blood pressure; C-IMT: common carotid artery intima-media thickness; EPCs: endothelial progenitor cells; FMD: flow-mediated dilation; PP: pulse pressure; PWV: carotid-femoral pulse velocity; RHI: reactive hyperemia index; sRAGE: soluble receptors for AGEs; T1DM: type 1 diabetes mellitus; WS: local carotid wave speed.

## Competing interests

Paolo Salvi, MD, PhD, is consultant to Diatecne for PulsePen

Michaela Kozakova, MD, PhD, is consultant for Esaote SpA

All other authors declare no competing interests.

## Authors' contributions

CP participated to study design, interpretation of data and writing final manuscript; MK contributed to analysis and interpretation of data, and revised the final manuscript; CM was responsible for acquisition and analysis of ultrasound and pulse wave velocity data; LG was responsible for selecting the T1DM population and performing and analyzing Endopat studies; MCB was responsible for EPCs determination; PS_1 _selected control population and contributed to the quality control of the overall database; FM performed AGEs, RAGEs, and adiponectin determination and analysis; PS_2 _contributed to the PulsePen acquisitions and data analysis and interpretation; AB revised the final manuscript and gave final approval; GS revised the final manuscript and gave final approval; RDS contributed to study design and interpretation and quality control of EPCs data; GF conceived the study, drafted a preliminary manuscript, and gave final approval of the version to be published.

All authors read and approved the final manuscript.
